# Step-wedge cluster-randomised community-based trials: An application to the study of the impact of community health insurance

**DOI:** 10.1186/1478-4505-6-10

**Published:** 2008-10-22

**Authors:** Manuela De Allegri, Subhash Pokhrel, Heiko Becher, Hengjin Dong, Ulrich Mansmann, Bocar Kouyaté, Gisela Kynast-Wolf, Adjima Gbangou, Mamadou Sanon, John Bridges, Rainer Sauerborn

**Affiliations:** 1 Department of Tropical Hygiene and Public Health, University of Heidelberg, Germany; 2 Health Economics Research Group (HERG), Brunel University, UK; 3 Institute for Medical Informatics, Biometrics, and Epidemiology, University of Munich, Germany; 4 Centre National de Recherche et de Formation sur le Paludisme, Ouagadougou, Burkina Faso; 5 Direction des Etudes et de la Planification, Ministère de la Santé, Ouagadougou, Burkina Faso; 6 Centre de Recherche en Santé de Nouna, Nouna, Burkina Faso; 7 Bloomberg School of Public Health, Johns Hopkins University, USA

## Abstract

**Background:**

We describe a step-wedge cluster-randomised community-based trial which has been conducted since 2003 to accompany the implementation of a community health insurance (CHI) scheme in West Africa. The trial aims at overcoming the paucity of evidence-based information on the impact of CHI. Impact is defined in terms of changes in health service utilisation and household protection against the cost of illness. Our exclusive focus on the description and discussion of the methods is justified by the fact that the study relies on a methodology previously applied in the field of disease control, but never in the field of health financing.

**Methods:**

First, we clarify how clusters were defined both in respect of statistical considerations and of local geographical and socio-cultural concerns. Second, we illustrate how households within clusters were sampled. Third, we expound the data collection process and the survey instruments. Finally, we outline the statistical tools to be applied to estimate the impact of CHI.

**Conclusion:**

We discuss all design choices both in relation to methodological considerations and to specific ethical and organisational concerns faced in the field. On the basis of the appraisal of our experience, we postulate that conducting relatively sophisticated trials (such as our step-wedge cluster-randomised community-based trial) aimed at generating sound public health evidence, is both feasible and valuable also in low income settings. Our work shows that if accurately designed in conjunction with local health authorities, such trials have the potential to generate sound scientific evidence and do not hinder, but at times even facilitate, the implementation of complex health interventions such as CHI.

## Background

Community health insurance (CHI) has been identified as a potentially valuable health financing alternative in low and middle income countries since, through the pooling of risks and resources, it promises to facilitate access to health services and increase financial protection against the cost of illness for vulnerable populations [1-5]. A number of recent reviews, however, highlight how scanty the evidence on the impact of CHI, defined in terms of its effect on health service utilisation and protection against the cost of illness, still is. They attribute this paucity largely to weaknesses in the design of the studies, mostly case studies and consultancy reports, which have so far been conducted to evaluate the schemes [6-9]. Specifically, key difficulties in assessing the validity of the information currently available on CHI are found to be related to the absence of baseline data and control groups, difficulties in sampling, the absence of control for confounding variables, weak sources of data, and lack of clearly defined outcome measures and indicators [6-9].

In this paper, we describe a step-wedge cluster-randomised community-based trial which we have been conducting since 2003 to accompany the implementation of a CHI scheme in a rural region of West Africa. The study aims to provide conclusive evidence on the impact of CHI on health service utilisation and protection against the cost of illness through the application of a trial design in line with the standards set by the Cochrane Collaboration through its Effective Practice and Organisation of Care (EPOC) group [10,11]. Given our innovative approach to evaluate the effects of a community-based intervention (CHI), we describe the trial design and discuss it in relation both to methodological considerations and to specific ethical and organisational concerns faced in the field. Our aim is to show that trials of this kind are feasible and that they can effectively be pursued to assess the impact of complex health policy interventions, such as health insurance, and not only, as traditionally done, specific disease control measures [12-14]. To our knowledge, only two studies have previously attempted to use cluster randomisation to evaluate the impact of health insurance programs in low income settings: one in India, described by Ranson and colleagues [15-17], and one in Mexico, described by King and colleagues [18]. Both studies, however, differ from the one described in this manuscript as they adopted a different methodology, i.e. not a step-wedge approach, and focused on assessing the impact of a specific set of additional interventions nested within the insurance program, rather than assessing the impact of the insurance scheme *per se*.

## Methods

### Context

The trial described in this article has been conducted since 2003 in the Nouna Health District, a region located in north-western Burkina Faso about 300 km from the capital Ouagadougou [19]. A demographic surveillance system (DSS) is operative in a sub-portion of the health district, covering a population of approximately 70,000 individuals who live in the catchment area of the Nouna Hospital, located in the district capital, and of six first-line health facilities located in the surrounding rural areas [20-22]. Plans to initiate a CHI scheme in the district followed the request of the Ministry of Health of Burkina Faso [23,23] and were preceded by extensive research exploring community perceptions of the quality of care, preferences and willingness to pay (WTP) for a benefit package, cost estimation analysis, risk perception and traditional networks of risk-sharing, health demand and health need assessment [24-29].

### Sample design

The area under demographic surveillance was subdivided into 24 rural (villages) and 9 urban (town of Nouna) clusters of approximately equal size. Clusters were purposely not defined according to the catchment area of the existing first-line health facilities (Centre de Santé et Promotion Social – CSPS) as their number changes continuously, but continues to be too low to allow for effective clustering. In 2003, the study area counted 4 CSPS; at the end of 2007, the number had increased to 7. In order to smoothen variation across clusters, small neighbouring villages were grouped to form one larger cluster [13,14,30,31]. This was done in the respect of geographical proximity, in order to avoid potential conflicts between neighbouring villages bound together by ethnic and kin ties, in case they should be offered insurance at different stages. The Burkinabè researchers assisted the German statisticians in the selection of the clusters to balance the need to abide to the sample calculations with the need to respect the local social context. This process was facilitated by the wealth of information available on each village thanks both to DSS data and to years of previous anthropological research experience in the study area. Given that clusters were purposely selected only within the existing DSS area in the Nouna Health District, where the CHI scheme was launched, the research team had no ambition to claim that these clusters represented a random sample from the population of all clusters nationwide.

**Figure 1:**
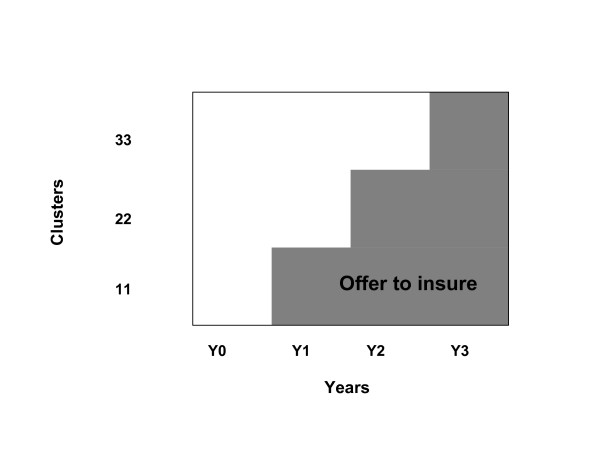
Step – wedge cluster- randomised community – based trial.

The 33 clusters were then randomized to intervention and control so that each year an additional 11 clusters were offered the opportunity to join the CHI scheme. The intervention was defined in terms of "offer to insure", given that enrolment in CHI was and continues to be voluntary. In 2004 (year 1), the first 11 clusters were offered insurance. In 2005 (year 2), both the first 11 clusters and an additional set of 11 clusters were offered insurance. In 2006 (year 3), the remaining 11 clusters were included in the intervention so that all 33 clusters are currently offered insurance (Figure 1). No further randomisation occurred within clusters as all households residing in one cluster were offered the possibility to join CHI at exactly the same time [13].

The information needed to evaluate the impact of CHI is collected through a household survey administered at least once a year to a statistically representative sample of all households residing in the DSS area. Given that the CHI is set to accept exclusively the enrolment of entire households, the sampling unit is the household, defined as the basic socio-economic unit within which individuals live together, share resources, and jointly satisfy their needs under the authority of the household head [32]. The DSS provided the sampling frame for the household survey. The sample size was estimated in advance to have a 90% power of detecting an increase in health service utilisation of one visit per year (Δ = 1) between insured and non-insured households, assuming a 2-sided type I error probability of 0.05 and, given the results of the prior WTP study [28], an enrolment rate of at least 50%.

It was estimated that had the study relied on randomisation of individual households (i.e. offering the opportunity to enrol to selected single households in the study area), a sample of 378 households would have been sufficient (189 per intervention arm) to detect a difference of one (Δ = 1) between insured and non-insured assuming a standard deviation of *σ *= 3 which appears a realistic value given a priori information. These calculations were based on the equation:

(1) $$n=\frac{{\left(u_{\alpha /2}+u_{\beta }\right)}^{2}*2\sigma^{2}}{\Delta^{2}}$$

where *u*_*α*/2 _and *u*_*β *_are the quantiles of the normal distribution and *σ *is the standard deviation of the number of visits per year.

Given cluster randomisation however, a design factor of 2.16 was applied to adjust for intra-cluster correlation coefficient (*ρ*) [14,31,33]. Using standard ANOVA calculations in fact [31], the analysis of a prior household survey conducted in the area in 2002 revealed that the intra-cluster correlation coefficient (*ρ*) for relevant variables (household socioeconomic status, proportion of household members reporting at least fair health, household spending on medical care) had a median of 0.04 (see Additional File 1). The number of households per cluster was set at m = 30. Thus, the basic sample size equation was modified as following:

(2) $$n=\frac{{\left(u_{\alpha /2}+u_{\beta }\right)}^{2}*2\sigma^{2}*\left(\left(1+(m-1\right)*\rho \right)}{\Delta^{2}}$$

to estimate that the minimum required sample should include 816 households distributed across 27 clusters. The minimum sample size was then increased to 990 households distributed across 33 clusters both to simplify the intervention by creating clusters in the respect of geographical proximity and feasibility, as explained in detail earlier, and to allow for a potential loss to follow up of 15%.

### Data collection and survey instruments

The survey collects information separately for each member of the households in the sample and covers all of the following areas: socio-demographic characteristics, income, expenditure, assets, illness reporting, and health care seeking behaviour, including health expenditure [32]. Two *ad hoc *modules, relevant to the evaluation of CHI, are administered to all people aged 15 and above. One, administered already at baseline in 2003 (year 0), assesses participation in traditional risk-sharing arrangements; and one, administered for the first time only at the end of the first enrolment campaign in 2004 (year 1), explores reasons motivating the decision to join or not to join the scheme. Household survey data are complemented with DSS data, providing information on village characteristics (e.g. village size, presence of a school, presence of a health facility), and with insurance data, providing information on the CHI campaign and overall village enrolment behaviour (e.g. number of sensitisation visits, sensitisation tools applied, enrolment status of village leaders). The quality of the data source is secured by a comprehensive control process, starting with the training and the direct supervision of field interviewers and ending with the verification of each questionnaire entered in the database [32].

### Data analysis

Given the study design, the impact of CHI is being assessed by comparing health service utilisation and protection against the cost of illness between insured and non-insured households, both within and across clusters. The impact of CHI on health service utilisation is being assessed by measuring differences in the number of curative visits to a health facility and delay to treatment given illness. In the short term, the impact of CHI on protection against the cost of illness is being assessed by measuring differences in catastrophic health spending. In the medium and long term, the impact of CHI on protection against the cost of illness can also be assessed by measuring differences in socio-economic status. Given that the intervention will stretch over a period of several years, the analysis needs to be adjusted for the time of the study allowing a better understanding of possible sources of bias.

Two important design issues need to be addressed in the analysis. First, statistical analysis ought to take into account that while the intervention is targeted at the cluster level, outcomes can only be assessed at the level of the household, thus requiring the application of hierarchical modelling techniques addressing the issue of intra-cluster correlation [14,31,34-37]. Preliminary analysis of the survey data has so far confirmed that the intra-cluster correlation coefficient (*ρ*) estimated to derive an adequate sample size calculations does in fact reflect the reality of the field trial [38]. Second, analysis ought to be corrected for the bias which arises from self-selection into the insurance scheme. Households with specific characteristics may in fact be more likely to purchase insurance than others [39], thus a direct comparison between insured and non-insured households would be likely to produce biased conclusions that may merely reflect the lack of initial comparability [40,41].

A number of methods have been developed to address selection bias in such an analysis [42-50]. Up to the third round of enrolment, propensity score techniques can be used to adjust for differences between insured and non-insured groups in a non-biased way by matching the self-selected treatment group, i.e. those having joined CHI, with an appropriate control group of people not having been offered CHI [40,41,51,52].

The information which has emerged from the analysis of enrolment behaviour following the end of the first round of enrolment has been used to inform the construction of propensity scores [39]. A first round of analysis on the effect of insurance status in improving access to care suggested a 40% increase in the number of outpatient visits and a 2% increase in the number of inpatient visits among insured people when compared to the uninsured people [53]. This analysis, which applied propensity score matching to account for potential selection bias into the scheme, used the Kernel matching method to estimate the average treatment effect on treated, i.e. the effect of insurance on access to care [53]. A limitation of such an analysis, however, is due to its ability to correct bias only on the basis of observable characteristics which determine self-selection. Thus, such an analysis is able to correct bias only partially.

After the third round of enrolment, once all households in all clusters have been offered the opportunity to join CHI, another set of techniques, primarily used in the analysis of observational data, ought to be applied to the trial data to assess the impact of CHI once the control group ceases to exist [54]. These techniques include behavioural models such as that of sample selection [42,44,45,47,48,50] and treatment effects and multivariate probit [43,55]. Analysing the impact of CHI from different techniques will provide robustness of the evidence imparted by the field trial.

## Discussion

EPOC guidelines value randomised controlled trials (RCTs) as the "gold standard" of study design. They recognise, however, that individual randomisation may not always be feasible and that, alternative study designs, such as cluster-randomised trials, may be better suited to provide adequate evidence on the impact of complex health interventions, including health system interventions [10,36,56]. In recent years, a number of researchers have been working on developing adequate methodologies to conduct cluster-randomised trials, reinforcing trust in how such trials can produce reliable evidence in situations when group randomization is to be preferred to individual one [14,30,31,33,34,37,57]. With the exclusion of the trial conducted by Ranson and colleagues in rural India [15-17] and of the trial conducted by King and colleagues in Mexico [18], however, research concerned with evaluating the impact of health financing interventions, including CHI, has mostly forgone the opportunity to take advantage of such methodological developments, thus failing to establish the evidence-base needed for health policy [6-9].

Our approach to the evaluation of CHI is therefore innovative and has the potential to produce sound evidence on the impact of such financing arrangement on health service utilisation and protection against the cost of illness. Furthermore, our research design and testimony of its feasibility under field conditions confirms the political acceptability of such trials already amply discussed by King and colleagues (2007)[18] and may serve as an example for the evaluation of other complex health system interventions, including other systems of health financing, adding to the very limited number of studies which have so far adopted comparably sound study designs [54,58-60].

In the specific case described in this article, a cluster-randomised community-based trial was preferred to a "simple" RCT since the latter would have posed both operational and ethical problems as single households within the same community would have had to be selected to receive the intervention, i.e. the offer to insure [14,31,36]. Contrary to the RAND experiment, which starting in the early 1970s randomised single American families to receive differential insurance coverage [54], communities in the study area would have objected individual randomisation, not only refusing to participate in the initiative, but potentially also withdrawing their trust from other activities managed by the health district and by the CRSN. This is most often likely to be the case faced by researchers working in societies with a collective orientation, whether in sub-Saharan Africa, Asia, or South America. In addition to limiting conflict both within and across communities, the adoption of cluster randomisation has minimised the researchers' interference with the work of the CHI management team, which has been able to manage the intervention (i.e. the implementation of the CHI scheme) directly. The research team has followed the work of the CHI management unit only from a distance, allowing for the initiative to be "owned" by the community. Qualitative research conducted in the area following the end of the first enrolment campaign showed that such an approach has substantially increased the acceptability of the CHI scheme as well as that of the accompanying study [61].

In addition, the step-wedge nature of the study has ensured that all communities would progressively be included the intervention. This represents a clear departure from previous studies, including the recent trial by Ranson and colleagues [15-17] and the one by King and colleagues [18], which also applied randomisation to understand the impact of health financing interventions. These studies in fact, entailed no possibility for the individuals or communities in the control areas to benefit from the intervention at a later stage [54,58-60]. What concerns were initially raised against the fact clustering entailed that some communities enjoyed the right to join the scheme earlier than others, were resolved through open discussion with the community in the light of the fact that, given the limited resources available, the CHI management team and the health district would inevitably have to carry out the intervention in a progressive manner regardless of the research. Thus, the application of a cluster-randomised community-based trial rather favoured the project implementation as it allowed random allocation of the intervention across communities, minimising possible grievances and complaints.

The step-wedge nature of the trial also allowed to minimise the spill over effect, as the incentive to migrate to a different area just to benefit from the intervention was counterbalanced by the fact that this very same intervention was going to reach the entire study area within a period of three years. The spill over effect was and continues to be further contained by the strict control on enrolment procedure which can be secured thanks to the availability of DSS data. During each enrolment campaign in fact, the CHI management unit checks on the DSS records that the people joining the scheme are actual residents of a given village. The DSS records as residents only those people who have resided for at least six months in a given village. Therefore, the CHI scheme runs no risk of enrolling people who have migrated temporarily with the only objective of joining the scheme.

The use of a cluster-randomised trial is often justified in terms of its ability to capture the overall effect of an intervention more adequately than a RCT [14,30,31]. Contamination across individuals is usually discussed in terms of *infectiousness *and *susceptibility*, often justifying the adoption of cluster-randomised trials for the evaluation of interventions targeting infectious diseases [14]. Although in the case of CHI, concepts of *infectiousness *and *susceptibility *do not apply as such, one needs to acknowledge the specific dynamic nature of the intervention. CHI in fact, is inscribed within the local social setting, with promotion campaigns delivering health messages to the entire population and with insured and non-insured households across clusters being bound together by ethnic and kin ties. Information is therefore inevitably shared both across insured and non-insured households and across clusters, possibly inducing some changes in the outcome variables regardless of insurance status. Unlike a RCT, a cluster-randomised trial allows for such *mass effects *to be captured [14,30,31], providing a more realistic estimation of the overall impact of the intervention on the community. Still, in order to limit the potential effect of cross-contamination across households and clusters on under-estimating the impact of the intervention, we grouped together several neighbouring villages to form a cluster, although we had no other means of specifically controlling for the proximity of control and treatment clusters. This has served the purpose of smoothing variation between clusters, thus increasing statistical efficiency in the analysis [14,30,31], while also facilitating the implementation of the intervention.

The study design described in this article allows to offset the limitations to the evaluation of CHI which have emerged in the literature [6-9]. The adoption of a cluster-randomised trial secures the recruitment of both cases, i.e. people who join CHI, and controls, i.e. people who do not join CHI, while analytical tools based on hierarchical modelling and propensity score techniques allow to minimise possible bias due to intra-cluster correlation and to the bias which arises from self-selection into the insurance scheme [13,14,34,40,41]. Furthermore, the field trial data will also allow other modelling techniques to be as applicable, thus offering a unique opportunity to compare results and validate evidence emerging from alternative analytical constructs. The adequacy of sampling was ensured by the fact that the sample selected for inclusion in the household survey took into account a design factor of 2.16 to compensate for cluster randomisation [14,30,31,33] and that preliminary analysis following the trial implementation confirmed that such design factor was suitable given the intra-cluster correlation coefficient detected on the field [38].

The wealth of information gathered through means of the household survey, the DSS, and the insurance records allows the statistical analysis to account for possible confounding factors both at the household and at the community level [13,36]. Having conducted a round of the household survey just before the launching of the insurance scheme ensures the availability of baseline data to be included in the statistical analysis.

It needs to be noted, however, that as the trial proceeds, the evaluation of the intervention is challenged by an enrolment rate which has been steadily increasing since the launch of the scheme (from 4.8% in 2004 to 8% in 2007), but which remains well below the expectations of the scheme initiators, although perfectly in line with most other experiences described in the literature [62]. The low enrolment rate has forced the research team to adapt the methodology originally envisioned *in itinere*, adopting methodological strategies which have proved to be more complex than what originally expected [53].

## Conclusion

Our experience conducting a step-wedge cluster-randomised community-based trial shows that the application of such studies is feasible to evaluate complex health interventions, even when working in resource-limited settings, and provides a unique opportunity to produce sound evidence for decision-making in health policy. In line with what reported by King and colleagues (2007)[18], our experience further shows that if accurately designed in conjunction with local health authorities, such trials do not to hinder the implementation of the intervention, but rather facilitate it with regard to some specific aspects. Given the scanty evidence currently available to adequately assess the impact of different health financing interventions, especially CHI [6-9], the research approach adopted by our team may be replicated in other settings as a means of contributing to evidence-based decision-making in health care. Similarly, our experience suggests that given their feasibility and their valuable potential to inform decision-making, researchers are therefore encouraged to use such trial designs and apply them to the study of other complex health policy interventions.

## Competing interests

The authors declare that they have no competing interests.

## Authors' contributions

All authors contributed to the conception and development of the study design, the review of the literature, and the revision of the final text. MDA drafted the manuscript with contribution from all authors. MDA and SP were the primary responsible authors for the review of the literature. RS, HD, HB, UM, and BK developed the initial idea for the study design. HD, UM, HB, and GKW were responsible for the statistical work which guided choices regarding the study design and the sample size. All authors contributed to the design of the data collection tools. HD, MDA, AG, BK, and MS were jointly responsible for implementing the field trial. SP, MDA, JB, and RS developed the conceptual models and the methodology to be applied to the analysis of the trial data. As first and corresponding author, MDA had full access to all the information on the study and had the final responsibility on the decision to submit for publication.

## Supplementary Material

Additional file 1 **Intra-cluster correlation coefficient calculations.** Details of the intra-cluster correlation coefficient calculations used to derive the sample size. Click here for file
