# Supplementary material for: Step-wedge cluster-randomised community-based trials: An application to the study of the impact of community health insurance
Source: Health Res Policy Syst. 2008 Oct 22;6:10. doi: 10.1186/1478-4505-6-10 (PMC2583992; doi:10.1186/1478-4505-6-10)
Supplement: Additional file 1 — Intra-cluster correlation coefficient calculations. Details of the intra-cluster correlation coefficient calculations used to derive the sample size. [file 1478-4505-6-10-S1.pdf]

Household survey data set: all: 777 households
Income < 10000 deleted: 717 households
(1 household with income=15476750 was deleted)

Results of linear regression

$Y_{ij} = \alpha_i + \varepsilon_{ij}$ i=1,…..,25 (k= 25 villages and respective sectors in Nouna town)
j=1,….., i_j (ca. i_j = 20-58 households)

$$\rho = \frac{ms_b - ms_w}{ms_b + (m_0 - 1)ms_w}$$

ρ = intra class correlation coefficient
R**2 = reliability coefficient
m_0 = mean cluster size (here m_0 = 31 = mean number of households per village)
ms_b and ms_w = mean square errors between and within cluster from standard ANOVA

	Y-Variable	data set	R**2	ρ
1	Income	< 10000 delete	0.09	0.066
2	Income	All	0.09	0.06
3	Log (10) [income]	< 10000 delete	0.09	0.069
4	Log (10) [income+1]	All	0.07	0.04
5	Income/household size	< 10000 delete	0.09	0.066
6	Log (10) [income+1]/household size	All	0.05	0.022
7	Total number of people in HH reporting at least fair health /household size	All	0.05	0.0186
8	Total household expenditure on drugs	all , only: 290 HH	0.08	-0.0075
9	Log(10)[Drugs+1]	all , only: 290 HH	0.13	0.054
10	Log (10) [Income+1]/household size<br>Stratified rural/urban	rural: 453 HH<br>all<br>urban: 287 HH	0.03<br>0.04	-0.0068<br>0.017
mean				0.036
median				0.04
